# Effects of traditional fitness qigong exercise on frailty status and overall well-being in frail or pre-frail patients: a systematic review and meta-analysis

**DOI:** 10.3389/fmed.2025.1619729

**Published:** 2025-07-14

**Authors:** Weijing Sun, Lingling Li, Zhenzhen Zhang, Xiuli Duan, Juanjuan Wei, Kim Lam Soh

**Affiliations:** ^1^Department of Nursing and Rehabilitation, Faculty of Medicine and Health Sciences, Universiti Putra Malaysia, Serdang, Selangor, Malaysia; ^2^Department of Nursing, Faculty of Medicine, Bozhou Vocational and Technical College, Bozhou, Anhui, China; ^3^Department of Nutrition, Bozhou People’s Hospital, Bozhou, Anhui, China; ^4^Department of General Medicine, The Third People’s Hospital of Hefei, Hefei, Anhui, China

**Keywords:** frailty, pre-frailty, traditional qigong exercise, older adults, elderly care

## Abstract

**Objective:**

This study aimed to assess the effectiveness of traditional Chinese Fitness qigong exercises (TFQs) in enhancing outcomes such as frailty status, physical function, psychological well-being, cognitive function, negative emotions, and sleep quality for older adults with frailty or pre-frailty.

**Methods:**

We conducted searches in Embase, Cochrane Library, PubMed, Web of Science, China National Knowledge Infrastructure (CNKI), Wanfang, Chinese Technical Periodicals (VIP), and Google Scholar from their inception to 30th July 2024. Two reviewers independently selected and screened the papers, extracted the data, and assessed the bias risk. We used Review Manager 5.4 for data analysis, pooling the mean difference (MD) or standardized mean difference (SMD) through either random-effects or fixed-effects meta-analysis, depending on the level of heterogeneity.

**Results:**

Eighteen randomised controlled trials were included. A total of 15 studies, with 899 participants and versatile assessment tools (Fried Phenotype, Frailty Index, Tilburg Frailty Indicator), demonstrated the effectiveness of TFQs in reducing frailty. Subgroup analysis revealed significant improvements using FP scores (MD = −1.83, 95% CI: −2.09, −1.5), TFI (MD = −1.08, 95% CI: −1.22, −0.94), and FI (MD = −0.04, 95% CI: −0.08, −0.0). Physical performance improved significantly (MD = −0.72, 95% CI: −0.88, −0.57), while daily living activities showed no statistically significant changes (SMD = −0.20, 95% CI: −0.52, 0.11). Balance ability, including dynamic (MD = −2.55, 95% CI: −2.88, −2.22) and static balance (MD = 3.51, 95% CI: 3.00, 4.02), demonstrated notable enhancements. Grip strength increased significantly (SMD = 0.76, 95% CI: 0.51, 1.02), while gait speed improvements were more consistent in shorter walking distances (4.5 m: MD = − 1.44, 95% CI: −1.66, −1.22) than longer ones. Cognitive function (MD = 2.34, 95% CI: 0.35, 4.33) and sleep quality (SMD = 1.28, 95% CI: −1.69, −0.87) also exhibited substantial improvements. Quality of life (SMD = 1.17, 95% CI: 0.92, 1.42) and reductions in negative emotions (SMD = −0.79, 95% CI: −1.36, −0.22) were statistically significant.

**Conclusion:**

Traditional Chinese Fitness qigong exercises (TFQs) significantly improved multiple outcomes for frail or pre-frail older adults, including frailty levels, physical performance, grip strength, balance ability, cognitive function, sleep quality, and quality of life. These results suggest that TFQs protect frailty or prefrail older adults.

**Systematic review registration:**

https://www.crd.york.ac.uk/PROSPERO/view/CRD42024555719; Identifier: CRD42024555719.

## 1 Introduction

The aging of the global population has brought increased attention to health problems affecting older adults (1). Frailty represents a significant issue among these concerns, not only impacting the well-being of this demographic (2) but also acting as a key barrier to healthy aging due to its multidimensional nature involving interconnected physical, cognitive, and psychosocial declines (3). Such adverse outcomes include functional decline, disability, falls, hospitalisation, and mortality (4). This condition not only influences physical health but also demonstrates strong connections to mental health and overall quality of life (5). Interventions aimed at preventing, delaying, or reversing frailty’s progression are crucial due to its significant effect on older adults (6). Among the most promising strategies available, exercise interventions stand out for their effectiveness in mitigating frailty’s effects (7). For frail older adults, balance and aerobic exercises are frequently recommended specifically to enhance physical capacity and general health (8). Traditional fitness qigong exercises (TFQs), as integral components of Traditional Chinese Medicine (TCM), combine physical activity with breath regulation and meditative focus. Evidence suggests they may counteract frailty through neuromuscular enhancement, autonomic regulation, and psychological resilience (9, 10). Styles such as Tai Chi, Baduanjin, Yi Jinjing, and Wuqinxi belong to this category, each with unique characteristics. Existing meta-analyses offer suggestions that these traditional Chinese exercises might improve frailty status among older adults (11). However, three critical gaps limit current evidence: 1. Population specificity: Prior syntheses rarely required validated frailty assessments (e.g., Fried criteria), instead including broad populations with unconfirmed frailty status (12); 2. Intervention heterogeneity: TFQs are frequently pooled with dissimilar mind–body practices (e.g., yoga) in analyses (13); 3. Outcome fragmentation: Few reviews concurrently assess physical, cognitive, and psychological domains with validated tools (14). This fragmented approach conflicts with the World Health Organization’s (WHO) Integrated Care for Older People (ICOPE) framework, which mandates integrated management of intrinsic capacity (physical, cognitive, psychological) in frail older adults (15). To address these gaps, we:1. Exclusively include trials with validated frail/pre-frail diagnoses;2. Restrict interventions to defined TFQs (e.g., Tai Chi, Baduanjin);3. Systematically synthesize outcomes across five domains: frailty status, physical capacity, cognitive function, emotional well-being, and quality of life. While broad questions pose methodological challenges, our design provides incremental value through population specificity, intervention purity, and multidimensional synthesis (16). This approach allows direct application to Traditional Chinese Medicine (TCM)-based frailty management strategies. The objective guiding this study involves systematically evaluating TFQ effects on frail and pre-frail older adults, with emphasis on their frailty status, physical capabilities, cognitive performance, quality of life, emotional state, and sleep quality. *Our primary research question is thus posed: How does participation in traditional fitness qigong exercise affect frailty status, physical function, cognitive health, emotional well-being, and quality of life in older adults diagnosed as frail or pre-frail?*

## 2 Materials and methods

This systematic review and meta-analysis adhered to the recommendations of the Cochrane Handbook and the Preferred Reporting Items for Systematic Reviews and Meta-Analyses (PRISMA) (17). The protocol was registered *a priori* in the PROSPERO database (International Prospective Register of Systematic Reviews) under registration number CRD42024555719.

### 2.1 Search strategy

This study screened references using the PICO framework. The following databases were searched: PubMed, Embase, Cochrane Library, Web of Science, CNKI, Wanfang, VIP, and Google Scholar, covering the literature from the inception of each database to July 2024. We utilised Medical Subject Headings (MeSH) and their equivalents, alongside relevant text word terms, to ensure comprehensive coverage of the literature. A systematic literature search was conducted to identify prospective Qigong research articles, focusing specifically on frailty. Databases were searched utilising the keywords “frail*,” “elder*,” “older adult,” and “pre-frail*.” Employing a fuzzy matching method to account for variations in terminology, a subsequent full-text search incorporated the keywords: “Tai Chi,” “BaDuanJin,” “Qigong,” “Wu Qin Xi,” “Yi Jin Jing,” “traditional Chinese sports,” and “traditional Chinese exercise.” The search was confined to literature published in English and Chinese to capture the most relevant studies in these languages, with no date restrictions applied.

Besides, the search strategy for each database was optimized for sensitivity and comprehensiveness, including the title, abstract, and subject headings. Following the initial searches, duplicate entries were removed, and articles were screened according to pre-established inclusion and exclusion criteria.

To assess the comprehensiveness of the original search strategy and address potential limitations regarding frailty-related terminology raised during peer review, we conducted a supplementary search in PubMed on June 18, 2025. Broader conceptual terms related to frailty (e.g., “functional decline,” “geriatric syndrome,” “physical resilience,” and “frail elderly” [MeSH: NoExp]) were combined with predefined Qigong-related intervention terms. The search was restricted to studies published after the original cut-off date (July 1, 2024).

### 2.2 Eligibility criteria

The inclusion criteria were established using the PICO framework (Population, Intervention, Comparison, Outcomes) (18), as outlined in the following criteria were applied (Table 1).

**Table 1:** Population, intervention, comparator, outcomes (PICO) eligibility criteria.

Item	Content
Population (P)	Adults aged ≥ 60 years identified as frail using any validated frailty assessment tool [e.g., the Fried Frailty Phenotype (5), the Rockwood Frailty Index (27)] or explicit author-established criteria. Studies including participants who were pre-frail were also considered.
Intervention (I)	Chinese traditional fitness Qigong exercise (TFQs), including methods like Tai Chi, Baduanjin, Yi Jin Jing, and Wuqinxi.
Comparison (C)	The control group was defined as receiving usual care, routine care, or minimal contact without intervention or treatment components. Any head-to-head interventions were excluded.
Outcomes (O)	The outcomes include frailty status (assessed with a valid tool), physical ability (such as activities of daily living, balance tests, and walking ability), cognitive function, quality of life, emotional well-being, and sleep quality.

In addition to the PICO criteria, the following inclusion criteria were applied: 1. publication in English or Chinese; 2. use of a randomised controlled trials (RCTs) design. Studies were excluded if they met any of the following conditions: 1. absence of a transparent or verified definition of frailty for the study population; 2. incomplete statistical reporting resulting in questionable findings; 3. interventions other than TFQ methods as the primary treatment modality; 4. a mean participant age is below 60 years; 5. study design is classified as commentary, review, meta-analysis, Qigong protocol, or qualitative research.

### 2.3 Study selection, data extraction, and synthesis

Two evaluators (WJS, XY) independently screened titles and abstracts to identify articles necessitating full-text review. They acquired relevant publications and assessed these according to predefined eligibility criteria outlined in Table 1. Discussions were held to reconcile any discrepancies between reviewers, ensuring joint agreement on all decisions. Resolution of any remaining disputes involved consultation with a third expert reviewer. Data extraction was performed independently by two reviewers who utilised a standardized data extraction form created by the investigators. This form was structured to capture critical details, such as author information, publication year, study’s geographic location, the definition of frailty used, e.g., Fried Frailty Phenotype (FP) or Rockwood Frailty Index (FI), sample size, participant characteristics, details of intervention and control groups (including TFQ method duration and frequency), and outcome measures such as frailty status, quality of life, physical function (e.g., activities of daily living, walking ability, balance tests), cognitive function, sleep quality, and negative affect. The two reviewers cross-checked the extracted data, resolving any data extraction discrepancies through a similar consensus process.

Duplicate articles were identified and removed using Endnote 20 software following importation. Independent analysis of full-text articles by two reviewers identified and excluded duplicate publications. Articles outside the inclusion criteria or demonstrating methodological inconsistencies were excluded prior to the final analysis. We employed meta-analytical methods to synthesize the extracted data. A meta-analysis was performed when baseline and follow-up data on frailty status or other relevant outcomes were reported by two or more studies. Narrative summarization was used for data unsuitable for meta-analysis.

### 2.4 Risk of bias assessment

Assessment of the risk of bias in the included studies utilised the Cochrane Risk of Bias Tool (RoB 2.0) (19). Five specific domains were considered in this assessment: random sequence generation, allocation concealment, blinding of participants and personnel, incomplete outcome data, and selective reporting. For each domain, studies were categorized as exhibiting low, high, or uncertain risk of bias. Disagreements between the primary assessors were resolved through consultation with a third reviewer. We conducted sensitivity analyses focusing on studies with small sample sizes, missing data, or those identified with a higher risk of bias, aiming to further ensure the stability and robustness of the synthesis findings. Studies received a low overall risk of bias designation if they demonstrated low risk across three critical domains: random sequence generation, allocation concealment, and missing outcome data (20). High overall risk of bias was attributed to all other studies. Additionally, the GRADE (Grading of Recommendations, Assessment, Development, and Evaluation) framework was applied to evaluate the certainty of evidence for every outcome. Evidence quality was rated by this framework as high, moderate, low, or very low, considering factors such as study limitations, result consistency, indirectness, imprecision, and publication bias.

### 2.5 Statistical analysis

Statistical analyses were performed using RevMan 5.4 software provided by the Cochrane Collaboration, Oxford, United Kingdom. For continuous data, we calculated a weighted mean difference (MD) when studies employed identical measurement instruments; a standardized mean difference (SMD) with a 95% confidence interval (CI) was calculated if different instruments were employed. The participant count was adjusted for studies featuring two exercise or control groups, while means and standard deviations remained unchanged. Assessment of heterogeneity involved with the I^2^ statistic. Homogeneity, indicated by *p* > 0.1 and I^2^ < 50%, prompted the use of a fixed-effects model. In comparison, a random-effects model was applied when *p* < 0.1 and I^2^ ≥ 50%. Subgroup analyses consistently utilised a random-effects model. Exploration of potential sources occurred in instances of significant heterogeneity (*p* < 0.1). Descriptive analyses were performed when suitable. The robustness of the results was examined through sensitivity analyses, which concentrated on factors including small sample sizes, missing data, and studies possessing a higher risk of bias.

## 3 Results

### 3.1 Search summary

**Figure 1:**
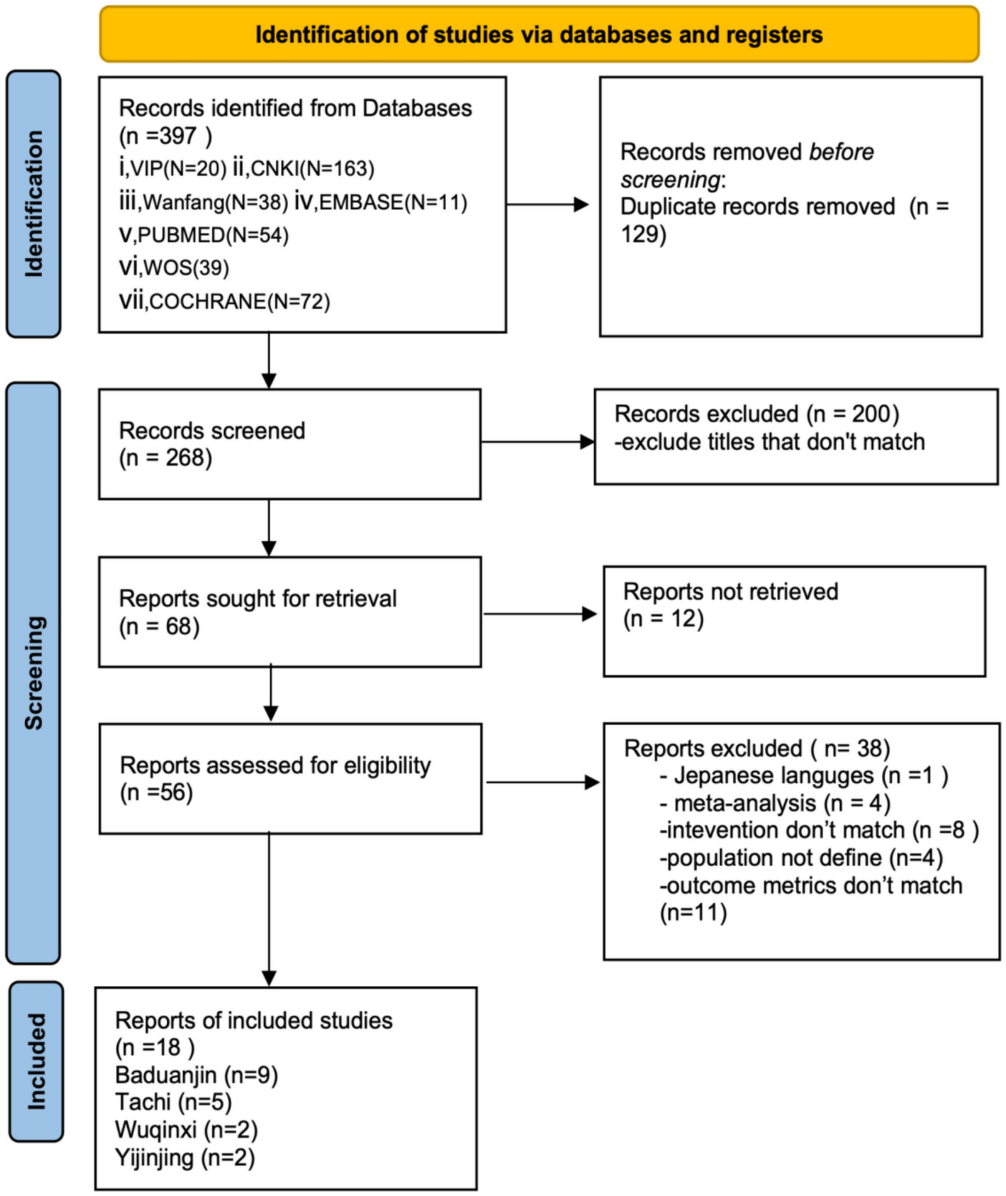
PRISMA flowchart.

An initial pool of 397 relevant articles was examined. After removing 129 duplicates, 200 irrelevant articles were excluded based on a preliminary screening of titles and abstracts. Further, a full-text review resulted in the exclusion of 38 additional articles. Finally, eighteen articles were retained for analysis (Figure 1). The supplementary search identified one additional article. Upon title and abstract screening, the study was excluded because it did not meet the inclusion criteria—it was a meta-analysis on general exercise interventions and did not specifically address Qigong or frailty/pre-frailty. No additional eligible RCTs were identified, supporting the comprehensiveness of the initial search strategy.

### 3.2 Assessment of risk of bias in individual studies

**Figure 2:**
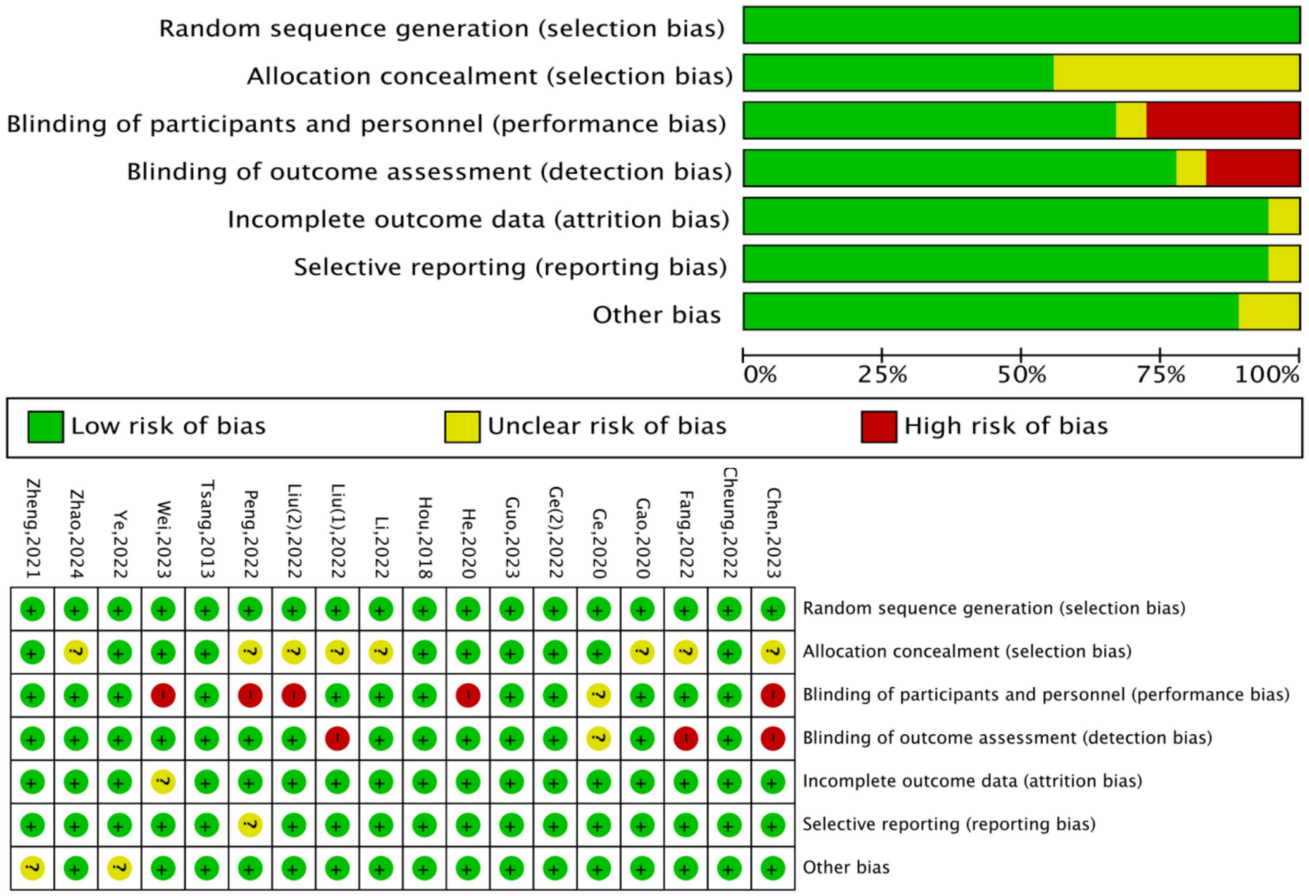
Risk of bias assessment: graphical representation and summary matrix.

A total of eighteen randomised controlled trials were evaluated using the Cochrane RoB 2.0 tool, with comprehensive visualization in Figure 2. Thirteen studies (72.2%) received a low-risk assessment concerning random sequence generation and allocation concealment, reflecting well-designed randomisation methods (e.g., computer-generated sequences) and allocation concealment strategies (e.g., sealed envelope methods). Clear descriptions of these methods were provided by studies such as Chen (21) and Zhao (22), as examples. In comparison, a few studies, e.g., Fang (23), were classified as unclear risk because detailed randomisation procedures were absent. Significant concerns emerged in blinding domains: Twelve studies (66.7%) had unclear risk due to undocumented blinding procedures [e.g., Ge (24)], while four studies (22.2%) were high risk [e.g., Hou (25); Tsang (26)] due to absent blinding, introducing performance/detection bias. Minimal risk was associated with incomplete outcome data and selective reporting; fifteen studies (83.3%) demonstrated low risk here owing to complete data reporting. Additionally, identification of independent methodological flaws did not occur in other bias domains, leading to a low-risk rating for all studies in these areas (Figure 2).

### 3.3 Characteristics of studies, outcome measurement

Table 2 presents the essential characteristics of the included studies. The 18 RCTs included various types of TFQs, including Baduanjin, Tai Chi, Wuqinxi, and Yijin Jing. These Qigong exercises incorporate common elements such as slow, deliberate movements, regulated breathing, and meditative practices. Comparison interventions in the control groups included newspaper reading, light exercise, education, non-exercise activities, and usual care. Thirty measures were used to evaluate 1,339 older adults classified as frail or pre-frail (666 in the intervention group and 673 in the control group). Frailty status was assessed utilising five different instruments: the Fried Frailty Phenotype (FP), Frailty Index (FI), Tilburg Frailty Indicator (TFI), Fried Frailty Criteria, and Comprehensive Frailty Assessment Instrument (CFAI). The FP concentrates exclusively on physical function, whereas the FI and TFI employ a detailed approach to frailty assessment (27). Five dimensions of physical capacity were evaluated: 1. overall physical performance, 2. activities of daily living, 3. ambulation, 4. balance, and 5. grip strength. Researchers utilised the 36-Item Short Form Health Survey (SF-36), 12-Item Short Form Health Survey (SF-12), World Health Organization Quality of Life-BREF (WHOQOL-BREF), and Life Satisfaction Index A (LISA) to measure quality of life (QOL). Cognitive function was measured with the Mini-Mental State Examination (MMSE), and negative affect was assessed through the Geriatric Depression Scale (GDS). Sleep quality was measured utilising the Pittsburgh Sleep Quality Index (PSQI).

**Table 2:** Summary of qigong interventions described in the included studies for outcome of frailty.

Authors, year, and langugage	Sample size (*N*)		Mean age (year ± SD)		Study population	Intervention	Assessment tool	Duration	Control	Outcomes
	I	G	I	G						
Cheung, 2022, English (28)	15	13	69.2 ± 3.88	69.4 ± 2.66	Pre-frail/frail	Baduanjin	Fried frailty criteria	16 weeks	Light flexibility exercise	A/B,1.2a/D,f/E,a
Hou, 2018, Chinese (25)	36	35	82.86 ± 4.37	83.84 ± 3.38	Pre-frail/frail	Baduanjin	FP/FI	12 weeks	Resistance training	A,a.b/B,2b.3a.4a/C,a/D,a/E,a
Fang, 2022, Chinese (23)	22	22	72.14 ± 4.76	74.50 ± 4.02	Frail	Baduanjin	TFI	12 weeks	Light flexibility exercise	A,c/B,3a/D,e
He, 2020, Chinese (29)	30	30	69.58 ± 8.38	69.65 ± 7.92	Pre-frail	Baduanjin	TFI	2 weeks	Usual care	A,c/B,1.2c
Zheng, 2021, Chinese (30)	33	34	72.50 ± 6.10	71.00 ± 5.37	Frail	Baduanjin	TFI (Chinese version)	12 weeks	Usual care and education	A,c/B,3b.5/D,d
Gao, 2020, Chinese (31)	34	34	79.79 ± 4.18	78.88 ± 4.66	Pre-frail	Baduanjin	FP	12 Weeks	Light flexibility exercise	A,a/B,4a. 3b.4b.4c 0.5/D,c
Guo, 2023, Chinese (32)	33	35	78.06 ± 7.14	79.46 ± 5.77	frail	Baduanjin	FP	12 weeks	Usual care and light flexibility exercise	A,a/B,5/C,a/D,b/E,a/F,a
Chen, 2023, Chinese (21)	20	22	85.2 ± 3.0	85.3 ± 2.4	frail	Baduanjin	TFI (Chinese version)	12 weeks	Usual care and education	A,c/B,5/D,a/E,c/F,a
Liu(1), 2022, Chinese (33)	42	42	62.85 ± 6.84	61.28 ± 6.32	frail	Baduanjin	CFAI	24 weeks	Wuxing music	A,e
Liu(2), 2022, English (34)	67	68	80.75 ± 2.99	80.74 ± 2.82	frail	Tai Chi	FP	48 weeks	Usual care	A,a/B,3b/C,a/D,b
Ge(1), 2020, Chinese (24)	32	33	70.16 ± 5.40	72.91 ± 6.61	Pre-frail	Tai Chi	FP	8 weeks	Usual care non-exercise	A,a/B,4c.4d
Ge(2), 2022, English (35)	32	33	70.16 ± 5.40	72.91 ± 6.61	Pre-frail	Tai Chi	FI	8 weeks	Usual care	A,b/E,a
Ye, 2022, Chinese (36)	50	50	77.69 ± 4.51	76.25 ± 5.30	Frail	Tai Chi	TFI	12 weeks	Usual care	A,b/E,a/B,4a.4c/D,d
Li, 2022, Chinese (37)	27	30	82.87 ± 2.41	83.01. ± 2.82	Pre-frail	Tai Chi	FP	30 Weeks	Usual care	A,a/B,5.3b/D,a
Wei, 2023, English (38)	52	57	73.30 ± 3.89	70.74 ± 3.52	Frail	Wuqinxi	FP	24 weeks	Strength and endurance training	B,5.3a.3b.4a
Zhao, 2024, Chinese (22)	40	40	57.38 ± 9.89	54.08 ± 11.34	Frail	Wuqinxi	FP	12 weeks	Usual care	A,a
Tsang, 2013, English (26)	61	55	83.33 ± 6.30	84.85 ± 6.03	Pre-frail/frail	Yan Chai Yi JinTen Section	Frailty index	12 weeks	Newspaper reading	B,5.4a/C,b
Peng, 2022, Chinese (39)	40	40	72.12 ± 6.47	71.85 ± 5.73	Frail	Yijinjing	Clinical Frail Scale (CFS)	8 weeks	Non-exercise	B,b,4e

A, Frailty status: a. Fried Frailty Phenotype (FP), b. Frailty Index (FI), c. Tilburg Frailty Indicator (TFI), d. Fried Frailty Criteria, e. Comprehensive Frailty Assessment Instrument (CFAI); B, Physical ability: 1. Short Physical Performance Battery (SPPB), 2. Daily living ability, a. Modified Barthel Index (MBI), b. Activities of Daily Living (ADL), c. Barthel Index (BI), 3. Walking ability, a. 6-Minute Walk Test (6MWT), b. Gait speed, 4. Balance ability, a. Timed Up and Go (TUG) test, b. Five times sit-to-stand test (FTSST), c. One-Leg Stand Test (OLST), d. Functional Reach Test (FRT), e. Berg Balance Scale (BBS), 5. Grip Strength; C, Cognitive function: a. The Mini-Mental State Examination (MMSE); b. Lowenstein Occupational Therapy Cognitive Assessment-Geriatric (LOTCA-G); D, Quality of Life: a. Short Form Health Survey Questionnaire (SF-36), b. World Health Organization Quality of Life-BREF (WHQOL-BREF), c. Life Satisfaction Index A (LISA), d. 12-item Short Form Health Survey (SF-12), e. The Minnesota Heart Failure Quality of Life Questionnaire (MHFQOL), f. European Organization for the Research and Treatment of Cancer Quality of Life Questionnaire (EORTC QLQ-C30); E, Negative Emotion: a. Geriatric Depression Scale (GDS), b. Self-Rating Depression Scale (SDS), c. Hamilton Anxiety Scale (HAMA); F, Sleep Quality: a. Pittsburgh sleep quality index (PSQI).

### 3.4 Frailty status

**Figure 3:**
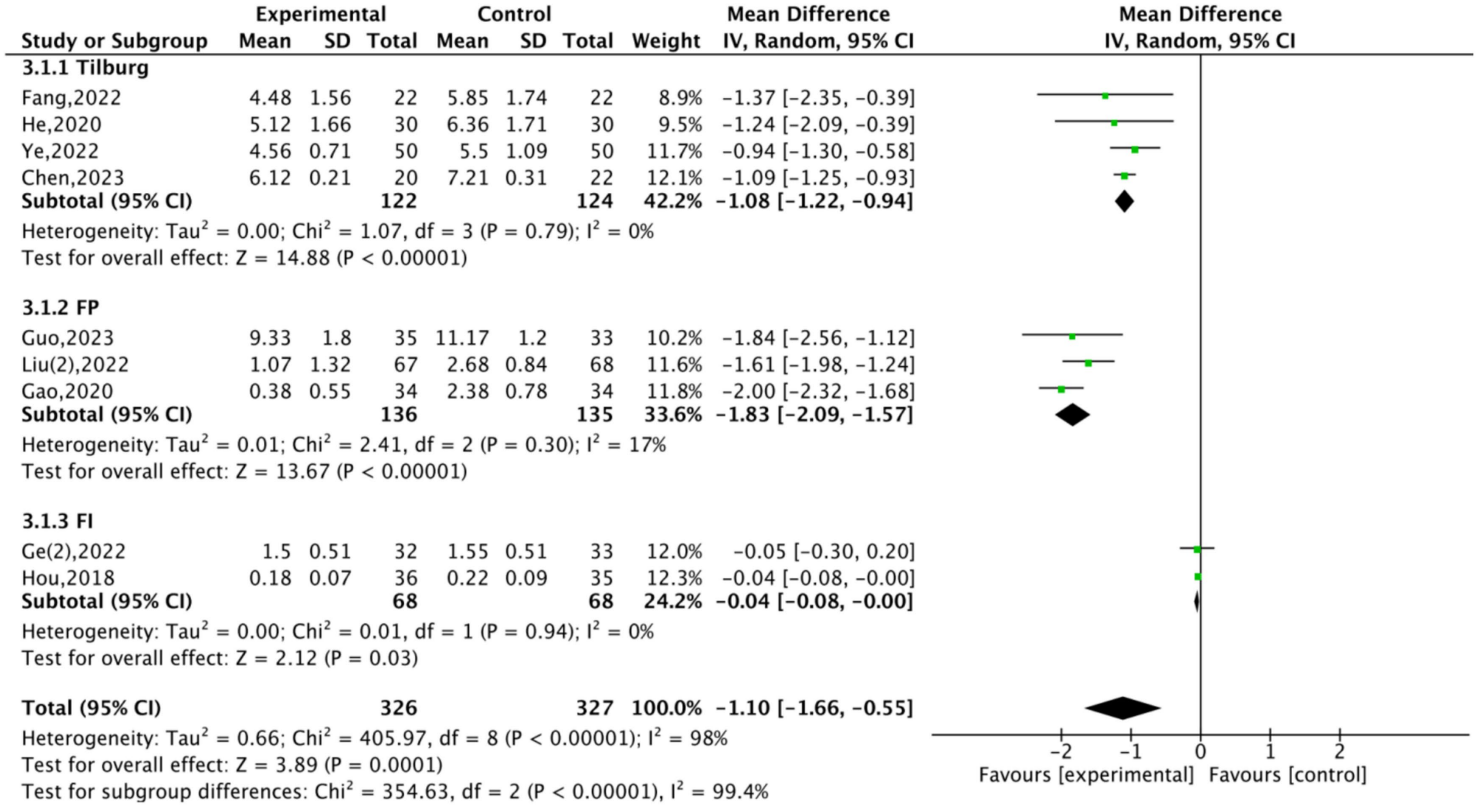
Forest plot of traditional fitness qigong exercise on frailty status.

Fifteen studies (21–25, 28–37) analysed the effects of Traditional Fitness Qigong exercises (TFQs) on frailty in older adults classified as frail or pre-frail. Nine of these studies, with 899 participants, were included in a meta-analysis. In this group, Hou (29) employed the FI and FP scores for assessment, while the remaining studies (22, 24, 31, 32, 34, 37) relied solely on FP scores. Cheung (28) and Liu (33) utilised the Fried frailty criteria and the CFAI. Studies (23, 29, 36) implemented the Tilburg Frailty Indicator TFI, and a Chinese version of TFI was utilised in studies (21, 30). To explore the effect of different assessment tools on outcomes, we performed a subgroup analysis based on the following factors: FP, FI, and TFI. Subgroup analysis allowed us to assess how each frailty assessment tool influenced the effectiveness of TFQ exercises. This analysis indicated studies utilising the FP scale (MD = −1.83; 95% CI: −2.09 to −1.57, *p* < 0.001, *I^2^* = 17%). Similarly, studies employing the TFI demonstrated a significant decrease (MD = −1.08; 95% CI: −1.22 to −0.94, *p* < 0.001, *I^2^* = 0%), as did those utilising the FI scale (MD = −0.04; 95% CI: −0.08 to −0.00, *p* < 0.001, *I^2^* = 0%). This indicates that the TFQ intervention is effective across different assessment tools, although the FP scale demonstrated the most substantial effect. All nine studies documented a significant decrease in frailty scores following the intervention (Figure 3). Moreover, four studies (22, 24, 30, 37) presented results utilising median and quartile data (Table 3). A consistent finding across these studies was that intervention groups performed better than control groups. Cheung (28) found that the frailty improvement rate was significantly higher in the intervention group (26.7%; 95% CI, 10.1 to 54%) than in the control group (15.4%; 95% CI, 3.7 to 46.0%). These results support the efficacy of TFQ for frailty reduction. Significant changes in frailty status for the intervention group were also reported by Liu (33); mean scores were (34.04 ± 3.21) for intervention versus (37.58 ± 3.47) for control (*p* < 0.001). To investigate the sources of heterogeneity, a meta-regression (Table 4) analysis was conducted. This analysis utilised assessment tools (FP, TFI) as covariates, while sample size and intervention duration served as controls. The analysis revealed that the type of assessment tool accounted for all the heterogeneity in the studies, indicating the tools’ influence on the outcomes. Revealed that assessment tools explained nearly all heterogeneity (*R*^2^ > 99%, *p* < 0.001). Residual heterogeneity was negligible (*I^2^* < 1%) Analysis showed the FP tool was associated with a significantly smaller effect size relative to the reference group (*β* = −1.90; 95% CI: −2.18 to −1.61; *p* < 0.001), which suggests this tool has a higher sensitivity to intervention effects. While the TFI tool yielded a moderate reduction (*β* = −1.03; 95% CI: −1.21 to −0.86; *p* < 0.001), it incompletely captured the intervention’s benefits. This underscores the critical importance of tool selection in frailty evaluation. Heterogeneity was not significantly influenced by either sample size (*β* = 0.0005; *p* = 0.861) or the duration of the intervention (*β* = 0.0059; *p* = 0.508). The robustness of these findings was confirmed via sensitivity analysis. This analysis involved sequentially excluding each study (leave-one-out method). Results from this process demonstrated the combined effect size remained stable (MD range: −1.80 to −1.90; all *p* < 0.001).

**Table 3:** Frailty status outcome.

Studies	Intervention [M (p25, p 75)]	Control [M (p25, p75)]	*p*-value
Ge (24)	1 (1.0, 2.0)	2.0 (1.0, 2.0)	<0.05
Li (37)	2 (2.0, 3.3)	2 (1.5, 2.5)	<0.05
Zhao (22)	2 (2.0, 3.0)	1 (0, 1.0)	<0.01
Zheng (30)	14 (13, 14)	11 (11, 12)	<0.01

**Table 4:** Meta-regression analysis result.

Variable	Regression coefficient (*β*)	Standard error (SE)	*p*-value	95% CI
Intercept (reference FI)	−0.14	0.12	0.240	[−0.37, 0.09]
FP Scale	−1.90	0.15	<0.001	[−2.18, −1.61]
TFI Scale	−1.03	0.09	<0.001	[−1.21, −0.86]
Sample size	0.0005	0.003	0.861	[−0.005, 0.006]
Intervention duration	0.006	0.009	0.508	[−0.012, 0.024]

### 3.5 Physical ability

Thirteen studies evaluated physical function using several different tools, including the SPPB for overall physical performance, ADL, MBI and BI for daily living abilities, 6MWT and gait speed for walking ability, grip strength for upper limb endurance, and the TUG along with the OLST and BBS for assessing dynamic and static balance, respectively.

#### 3.5.1 Physical performance

**Figure 4:**
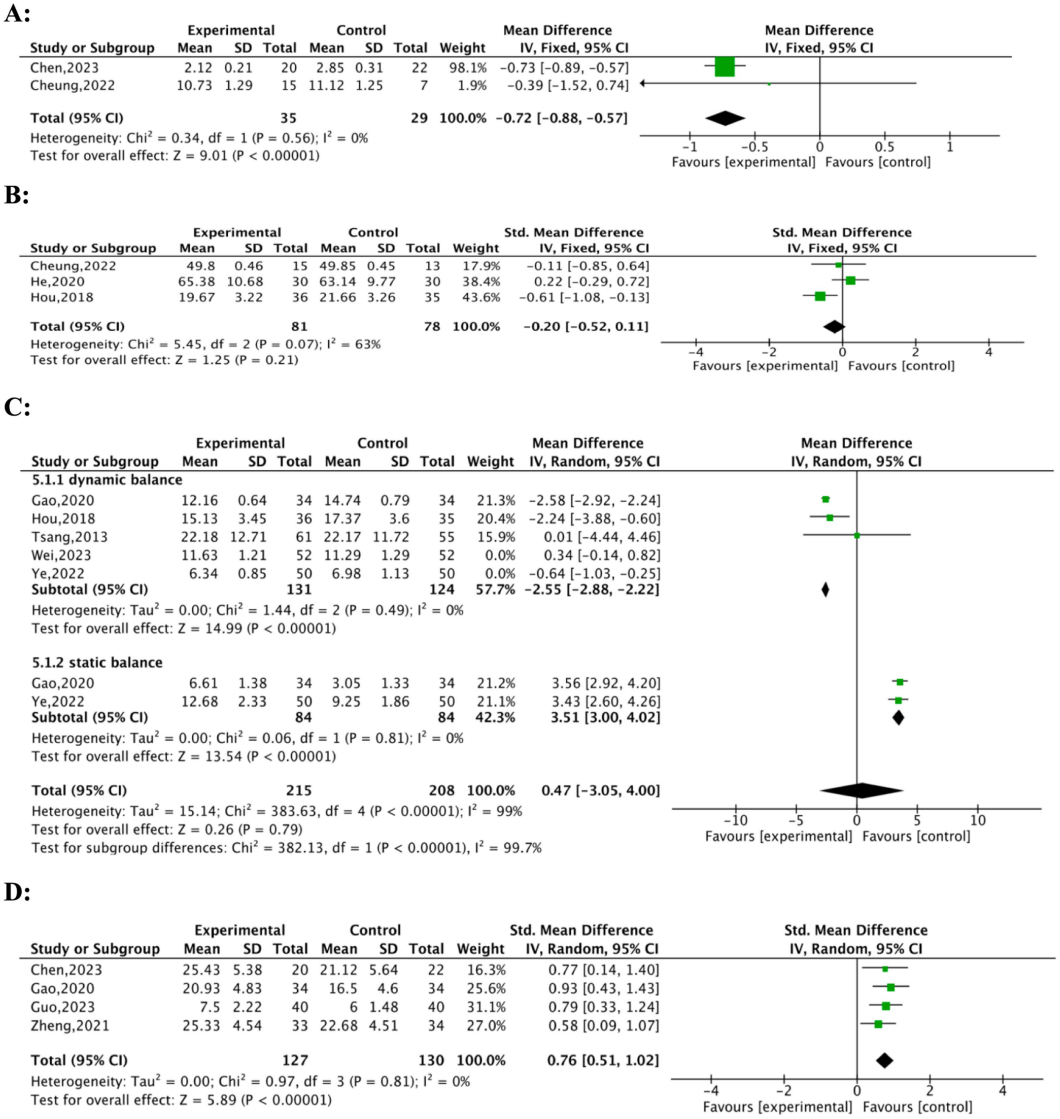
Forest plots of TFQs on physical performance, daily living activities, balance ability, grip strength. **(A)** physical performance, **(B)** daily living activities, **(C)** balance ability, **(D)** grip strength.

Three studies (21, 28, 29) evaluated the effects of interventions on physical performance. Meta-analysis (Figure 4A) indicated a significant effect favouring the experimental group (MD = −0.72, 95% CI: −0.88 to −0.57, *p* < 0.001, Z = 9.01). Heterogeneity was negligible (Chi^2^ = 0.34, df = 1, *p* = 0.56; *I^2^* = 0%). Chen (21) contributed 98.1% of the weight to the pooled estimate, while Cheung (28) contributed only 1.9%. These findings suggest a consistent advantage of the intervention compared to the control group. He (29) described significant results (10.96 ± 1.99, 9.38 ± 1.97 for the control, *p* < 0.01), although this study is not included in the final forest plot analysis shown in Figure 4A, a sensitivity analysis excluding He′s data confirmed low heterogeneity (*I^2^* = 0%). It maintained a significant pooled effect, enhancing the reliability of the result.

#### 3.5.2 Daily diving activities

Three studies (25, 28, 29) evaluated the effects of TFQs on the daily living activities of frail or pre-frail older adults, including 159 participants. Cheung (28) utilised the Modified Barthel Index (MBI), Hou (25) utilised the Activities of Daily Living (ADL) scale, and He (29) utilised the Barthel Index (BI) to measure daily living activities. The forest plot (Figure 4B) yielded a non-significant Standardized Mean Difference (SMD = −0.20, 95% CI: −0.52 to 0.11, *p* = 0.21, Z = 1.25), demonstrating no statistically significant overall impact on daily living activities. Moderate heterogeneity was observed (Chi^2^ = 5.45, df = 2, *p* = 0.07, *I^2^* = 63%), likely due to the different measurement tools employed. Notably, Hou (25) showed a significant improvement favouring the intervention (MD = −1.99, 95% CI: −3.58 to −0.40) and contributed the largest weight (43.6%). However, Cheung (28) (Weight = 17.9%, MD = −0.11, 95% CI: −0.85 to 0.64) and He (29) (Weight = 38.4%, MD = 2.24, 95% CI: −2.21 to 6.69) showed non-significant effects. While Hou (25) reported significant improvements in daily living activities, the overall evidence does not conclusively support a positive effect of TFQs on this outcome for frail or pre-frail older adults.

#### 3.5.3 Balance ability

We conducted a meta-analysis involving 423 participants to assess the impact of TFQs on balance ability in frail or pre-frail older adults; 215 participants were in the experimental group and 208 were in the control group. An initial pooled analysis incorporating five studies (25, 26, 31, 36, 38) indicated a significant improvement in balance performance (Figure 4C). However, this initial analysis revealed high heterogeneity (CI: −3.05 to 4.00, *p* = 0.79, Z = 0.26). Tau^2^ = 15.14; Chi^2^ = 383.63, df = 4, *p* < 0.00001; *I^2^* = 99%) and no significant overall effect (MD = 0.47, 95%Subgroup analysis revealed significant differences between dynamic and static balance outcomes (Test for subgroup differences: Chi^2^ = 382.13, df = 1, *p* < 0.00001; *I^2^* = 99.7%). Two studies were excluded: Wei (38), due to its significantly longer 24-week intervention duration compared to others, and Ye (36), as its data deviated as an outlier from the overall trend. Analysis was then optimized with the remaining three studies (25, 26, 31). This optimized analysis demonstrated a consistent and significant decrease in Timed Up-and-Go (TUG) test times (MD = −2.55, 95% CI: −2.88 to −2.22, *p* < 0.00001, Z = 14.99)with negligible heterogeneity (Tau^2^ = 0.00; Chi^2^ = 1.44, df = 2, *p* = 0.49; *I^2^* = 0%). supporting the robust nature of TFQs’ benefits for dynamic balance. Further insights were gained from subgroup analyses. Firstly, regarding static balance assessed through the One-Leg Standing Test (OLST), two studies (24, 36) reported significant improvements in the intervention group (MD = 3.51, 95% CI: 3.00 to 4.02, *p* < 0.00001; I*^2^* = 0%, Z = 13.54) with negligible heterogeneity (Tau^2^ = 0.00; Chi^2^ = 0.06, df = 1, *p* = 0.81; *I^2^* = 0%). Additionally, using the Berg Balance Scale (BBS), Peng (39) documented increased scores among the TFQs group (MD = 1.98, 95% CI: 1.16 to 2.79, *p* < 0.00001). Positive effects of TFQs were further corroborated by supplementary findings from studies excluded from the meta-analysis because their outcome formats were non-comparable. Ge (24), for example, employed the Functional Reach Test; results demonstrated significantly improved dynamic balance in the intervention group over controls (31.92 ± 5.3 vs. 22.34 ± 8.61; *p* < 0.01). Static balance outcomes from the same study (24), measured by the One-Leg Standing Test (OLST) and presented as medians with interquartile ranges, also favored the intervention group (intervention: 40.0 (21.6, 65.6) vs. control: 13.8 [8.5, 25.5]; *p* < 0.01). Independent support for these positive results came from Hou (25) through additional balance metrics (*p* < 0.01). Collectively, these results suggest TFQs consistently enhance both dynamic and static balance measures among older adults, showing minimal heterogeneity once methodological variations are considered.

#### 3.5.4 Grip strength

Seven studies (21, 26, 30–32, 37, 38) explored the effects of TFQs on grip strength in frail or pre-frail older adults. A meta-analysis of four studies (21, 30–32) involving 257 participants indicated that TFQs significantly enhanced hand grip strength in this demographic (SMD = 0.76, 95% CI: 0.51to 1.02; *p* < 0.01; *I^2^* = 0%, Z = 5.89, Figure 4D). A sensitivity analysis was performed to account for methodological variation that might affect the combined analysis. Therefore, we excluded the studies by Li (37), Wei (38), and Tsang (26) due to discrepancies. Specifically, Li′s study employed a more extended intervention (30 weeks), Wei’s control group utilised resistance training, and Tsang’s study included a significantly larger sample size. Removing these studies minimised heterogeneity and improved the consistency of the combined analysis. While excluded from the forest plot analysis, Li (37), Wei (38), and Tsang (26) individually reported significant gains in hand grip strength among frail or pre-frail older adults (24.2 ± 2.5, 17.3 ± 3.4, *p* < 0.01, 21.31 ± 3.44, 21.63 ± 3.26, *p* < 0.01, 15.63 ± 6.25, 14.62 ± 6.91, *p* < 0.01), supporting the primary finding.

#### 3.5.5 Walking ability

**Figure 5:**
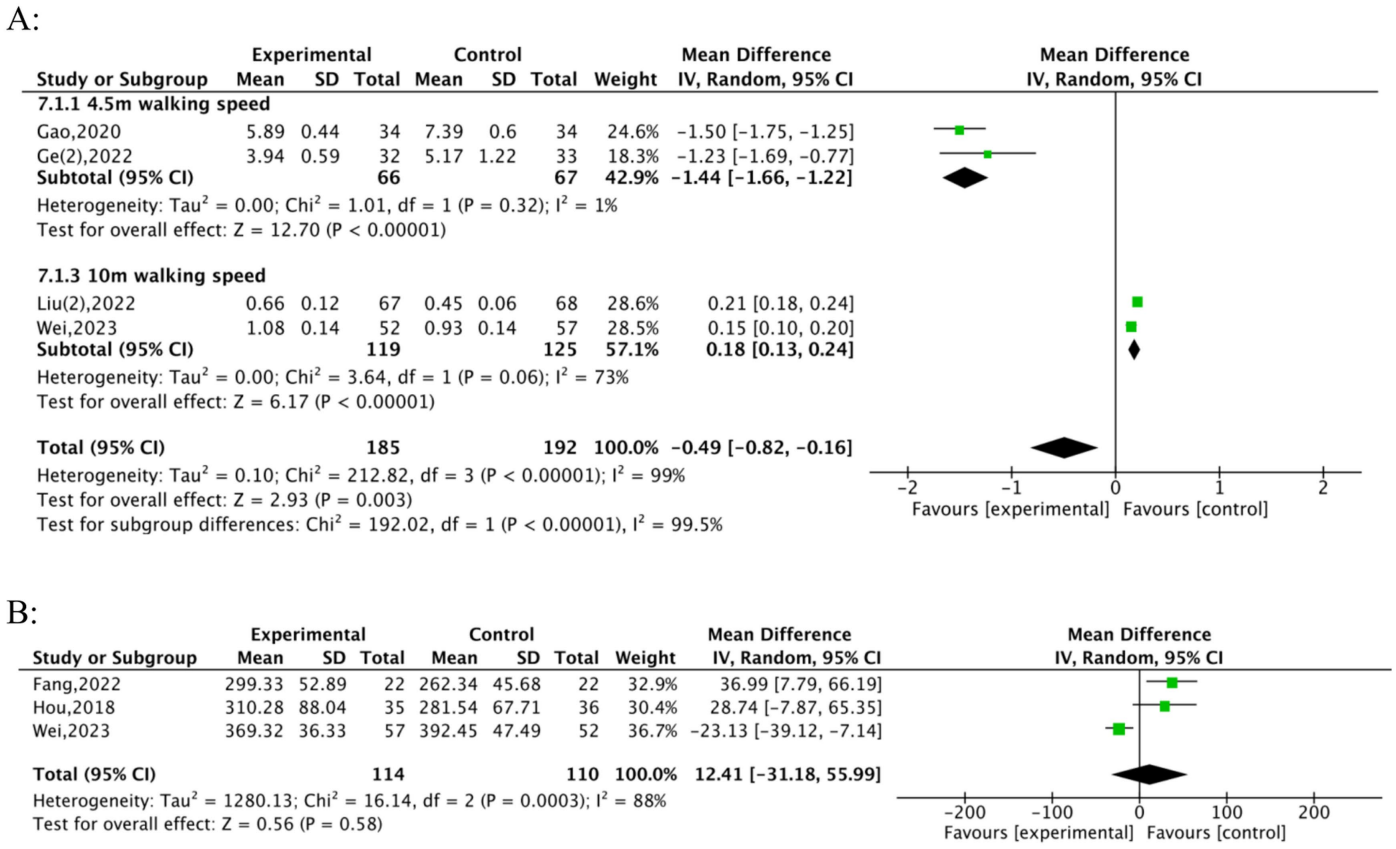
Forest plots of TFQs on walking ability. **(A)** Gait speed, **(B)** 6MWT.

Six studies (26, 30, 31, 33, 35, 37, 38) assessed the effects of TFQs on gait speed in frail or pre-frail older adults. The forest plot (Figure 5A) illustrates the heterogeneous effects of TFQs across two subgroups:4.5 m and 10 m walking speeds. Concerning 4.5 m Walking Speed: The studies by Gao (31) and Ge (35) demonstrated significantly faster walking speeds in the experimental group compared to the control, with a pooled mean difference (MD) of −1.44 (95% CI: −1.66to − 1.22; *Z* = 12.70, *p* < 0.00001). High consistency between these studies can be observed from low heterogeneity (*I^2^* = 1%, *p* = 0.32). Regarding the 10 m Walking Speed, both Liu (33) and Wei (38) also reported improvements in walking speed, with a pooled (MD = 0.18, 95% CI: 0.13 to 0.24; *Z* = 6.17, *p* < 0.00001). Differences in intervention protocols might be reflected by the moderate heterogeneity (*I^2^* = 73%, *p* = 0.06); one example is the longer duration used in Liu (33) compared to Wei (38). The overall pooled effect was significant but heterogeneous (MD = −0.49, 95% CI: −0.82 to −0.16; Z = 2.93, *p* = 0.003; *I^2^* = 99%), underscoring the critical impact of test distance on outcomes. Studies by Zheng (30) and Peng (39) were excluded. They assessed 6 m walking speed but demonstrated non-significant improvements (Zheng: 0.68 ± 0.07 vs. 0.66 ± 0.21, *p* < 0.01; Peng: 1.42 ± 0.03 vs. 1.11 ± 0.02, *p* < 0.01) and were incompatible with the framework for subgroup analysis. Li (37) offered data in the form of median and interquartile range (5.5 [5.0, 7.5], 10 [9.0, 11.3], *p* < 0.01), indicating that TFQs reduced the 6-meter walking time. Notably, improvements manifested differentially: Short-distance speed (4 m/6 m) showed consistent enhancements, while endurance outcomes (6MWT) exhibited non-significant pooled effects attributable to baseline imbalances and varied intervention durations. Four studies evaluated 6MWT performance. Li (37) was excluded from Figure 5B due to medians and interquartile ranges. Therefore, the analysis included three studies (23, 25, 38), comprising a total of 224 participants (114 experimental; 110 control). The meta-analysis did not find a statistically significant improvement in 6MWT performance (MD = 12.41; 95% CI: −31.18 to 55.99; Z = 0.56, *p* = 0.58). There was high heterogeneity associated with this result (*I^2^* = 88%, *p* = 0.0003, Figure 5B). Findings from Wei (38) were the primary source of this high heterogeneity; in that study, the control group exhibited superior baseline performance (mean 6MWT: 392.45 vs. 69.32 in the experimental group). It is possible that this baseline imbalance attenuated the intervention effect observed. Nevertheless, individual studies by Li (37) yielded promising results, indicating significant improvements in the intervention group compared to the control group (*p* < 0.01).

### 3.6 Cognitive function

**Figure 6:**
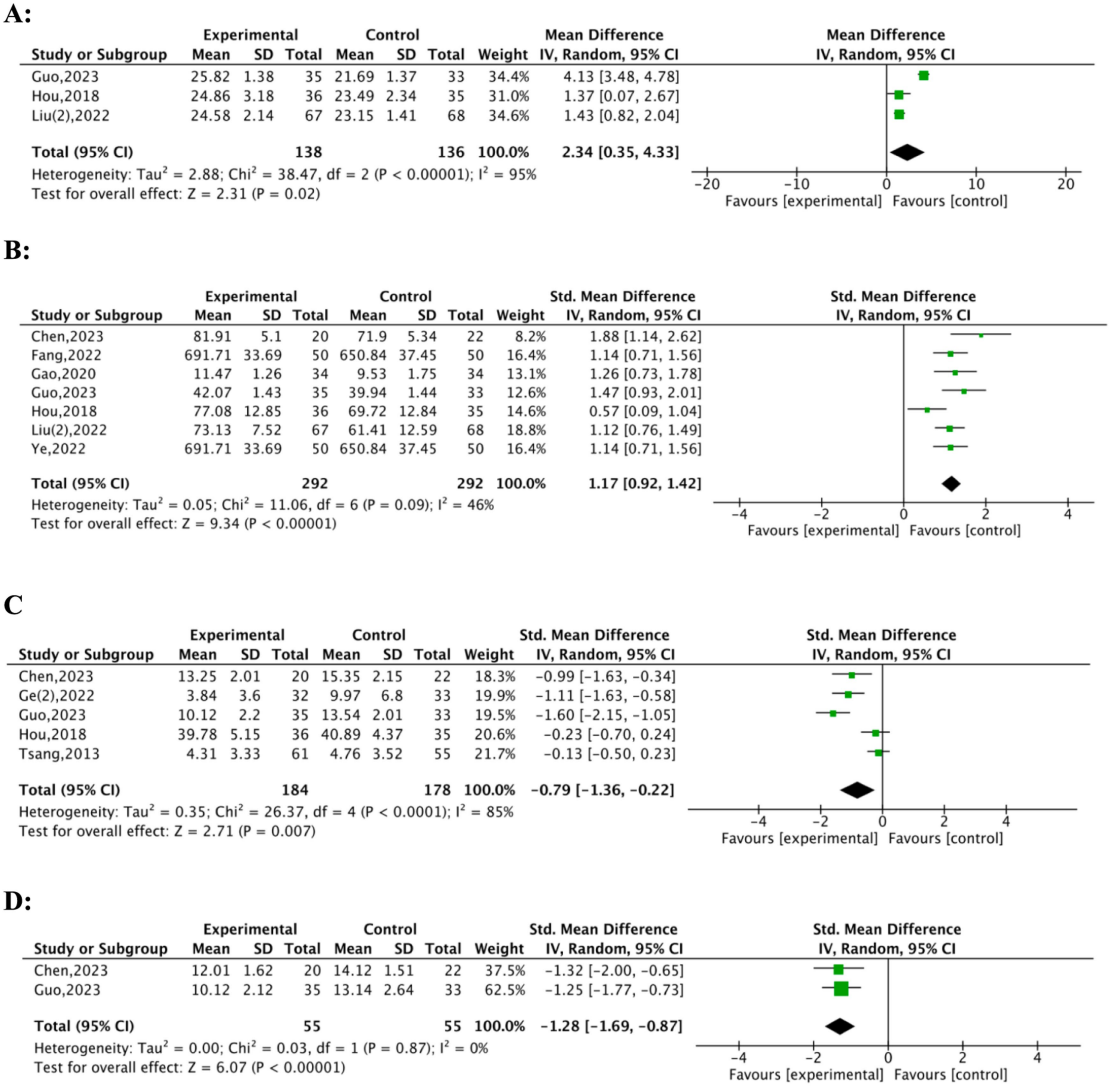
Forest plot of TFQs on cognitive function, quality of life, negative emotion and sleep quality. **(A)** Cognitive function, **(B)** quality of life, **(C)** negative emotion, **(D)** sleep quality.

Four studies (25, 26, 32, 33) reported the effects of TFQs on cognitive function in pre-frail or frail older adults. Significant improvements in cognitive function were observed in the experimental group compared to the control group (MD = 2.34; 95% CI: 0.35 to 4.33; Z = 2.3, *p* = 0.02, Figure 6A). The initial analysis, however, indicated high heterogeneity (Tau^2^ = 2.88; *I^2^* = 95%, Chi^2^ = 38.47, df = 2, *p* < 0.00001), Guo (32) was the primary heterogeneity source (Weight = 34.4%; MD = 4.13). Sensitivity analysis excluding Guo (32) resolved heterogeneity (*I^2^* = 0%) while maintaining significance [Hou (25): MD = 1.37, Liu (33): MD = 1.43; *p* < 0.05]. The resolution of heterogeneity suggests domain-specific benefits rather than generalized cognitive improvement. In addition, the stability of the results precluded the need for further subgroup analyses. Also, Tsang (26), utilising the LOTCA-G (a tool sensitive to executive planning) to assess cognitive function, reported significant enhancement (12.31 ± 1.1, 12.07 ± 2, *p* < 0.01). This further supports the conclusion that TFQs can enhance cognitive function in frail or pre-frail older adults, even with varied cognitive assessment tools.

### 3.7 Quality of life

Ten studies (21, 23, 25, 28, 30–32, 34, 36, 37) evaluated the effect of TFQs on the quality of life in frail or pre-frail older adults. These studies employed various assessment instruments: Chen (21) and Hou (25) utilised SF-36; Li (37), Zheng (30), and Ye (36) utilised SF-12; Gao (31) utilised LSIA; Guo (32) and Liu (34) utilised WHOQOL; Fang (23) utilised MHFQOL; and Cheung (28) utilised EORTC QLQ-C30. Seven studies (21, 23, 25, 31, 32, 34, 36), comprising 584 participants (292 experimental vs. 292 control), were finally included in a meta-analysis (Figure 6B) after excluding three studies [Cheung (28), Zheng (30), Li (37)] due to inconsistent data formats (median vs. mean ± SD reporting). TFQs significantly improved QoL (SMD = 1.17, 95% CI: 0.92–1.42; Z = 9.34, *p* < 0.00001) with moderate non-significant heterogeneity (*I^2^* = 46%, Tau^2^ = 0.05, Chi^2^ = 11.06, df = 6, *p* = 0.09). All individual studies favored intervention (SMD range: Chen = 0.81, Fang = 0.69, Gao = 1.88, Guo = 0.57, Hou = 0.77, Liu = 1.17, Ye = 0.69). Mixed results were reported in the excluded studies. Cheung (28) identified no significant improvement (SMD = 6.05 [−6.45, 18.56]; *p* = 0.744); whereas Zheng (30) and Li (37) reported significant benefits (Zheng: 52.70 [48.61, 58.33] vs. 44.44 [36.11, 50.00], *p* < 0.01; Li: intervention > control, *p* < 0.01). The primary analysis robustly supports the efficacy of TFQs for enhancing QoL, even considering these exclusions (*p* < 0.01 for post-intervention comparisons).

### 3.8 Negative emotion

Six studies (21, 24, 26, 28, 31, 32) analysed the effect of TFQs on the quality of life among frail or pre-frail older adults. Specifically, Ge (24), Guo (32), Cheung (28), and Tsang (26) utilised the GDS; Hou (25) utilised the SDS; and Chen (21) utilised the HAMD as assessment tools. Cheung (28) presented the median and interquartile range (0.13 [0.02, 0.24], 0.21 [0.10, 0.33], *p* < 0.05) and noted that TFQs could enhance the quality of life for frail older adults. A meta-analysis with 362 participants and drawing from five studies (21, 24, 26, 31, 32) indicated that TFQs significantly reduced negative emotion (SMD = −0.79, 95% CI: −1.36 to −0.22; Z = 2.71, *p* = 0.007); with high heterogeneity (*I^2^* = 85%, Tau^2^ = 0.35, Chi^2^ = 26.37, df = 4, *p* < 0.0001) (Figure 6C). The high heterogeneity observed derived primarily from the different scales utilised to evaluate the quality of life, precluding subgroup analysis.

### 3.9 Sleep quality

Two studies (21, 32) evaluated the effects of TFQs on sleep quality in frail and pre-frail older adults. The forest plot (Figure 6D) displays significant improvements in sleep quality in the experimental group relative to the control group. The pooled standardized mean difference (SMD = 1.28, CI: [−1.69 to −0.87], *I^2^* = 0%) indicates a large and statistically significant improvement (*Z* = 6.07, *p* < 0.00001). These findings offer strong evidence supporting the beneficial impact of TFQs on sleep quality in this population.

## 4 Sensitivity analyses

Due to the limited sample size per subgroup (fewer than 10), funnel plot analysis was not conducted. However, we performed sensitivity analyses to ensure the robustness and reliability of our findings. Transforming the fixed-effect model to a random-effects model and sequentially omitting individual studies did not significantly affect the results of the sensitivity analyses. By concentrating on dynamic balance, static balance, grip strength, ambulation, and cognitive function, we enhanced the accuracy of our pooled effect estimates, minimising the effect of heterogeneity.

## 5 Discussion

A meta-analysis of 18 randomised controlled trials offers compelling evidence that TFQs practices, including Baduanjin, Taichi, Wuqinxi, and Yijinjing, confer improvements in various dimensions of health among frail and pre-frail older adults. This study considered frailty status, physical capacity (including physical performance, activities of daily living, grip strength, ambulation, and balance), cognitive function, psychological performance, and intervention parameters (duration, frequency, location, and form). Our results demonstrate that diverse forms of Qigong yield significant positive effects; however, the underlying mechanisms and specific mechanisms vary depending on the health indicator assessed.

### 5.1 Frailty status

The results indicate significant improvements in frailty status among frail and pre-frail older adults following TFQ interventions. Meta-analytic results indicate a significant post-intervention decrease in frailty scores, consistently observed across multiple scales. This aligns with prior research indicating that traditional Chinese exercise can enhance balance, increase muscle strength, and reduce fatigue (40). Significantly, our meta-regression analysis indicated that the assessment tool type fully accounted for between-study heterogeneity (*R*^2^ = 100%). Figure 3 and Table 4 jointly provide critical insights: (1) FP’s physical focus yielded larger reductions (MD = −1.83) than TFI (MD = −1.08) or FI (MD = −0.04), and (2) meta-regression confirmed FP’s superior sensitivity (*β* = −1.90 vs. *β* = −1.03 for TFI). However, this specific finding warrants cautious interpretation. This statistical artifact might have been driven by extreme collinearity between covariates (like the FP vs. TFI scales) or potentially by overfitting due to outliers (such as the large effect size MD = −1.61 in Liu (34)). The dominant influence of assessment tools certainly highlights their methodological impact. Nevertheless, the almost zero residual heterogeneity (*I^2^* = 0.33%, Table 4) implies that the current conclusions could be limited by the narrow range of scales used and the small sample sizes in the included studies. Besides, improvements in TFI scores suggest that TFQs may contribute to better mental health and increased social interaction. The balance exercises and deep breathing incorporated in TFQs may improve vestibular function and vagal tone, thus lessening frailty through integrated mind–body mechanisms (41). As TFQs are frequently practised in group settings, they may promote social connections and enhance social support among older adults. However, differences in improvement were found across assessment tools. Improvements measured with the FP scale were more significant than the FI and TFI, potentially attributable to the FP scale’s emphasis on the physical aspects of frailty (42). Subgroup analyses further measured this divergence. Robust effects were apparent for the FP scale (MD = −1.46, 95% CI [−2.30 to −0.61]). Outcomes based on the TFI, conversely, showed uncertainty (CI spanning zero). This highlights that larger samples are necessary to validate multidimensional assessment tools. The significant reduction in FP scores (MD = −1.83) may reflect the direct effects of Qigong on physical strength, whereas the more modest change in FI scores (MD = −0.04) suggests that Qigong’s impact on psychological function may be less significant, potentially requiring longer interventions for detectable effects (43). This observation is consistent with prior research indicating improvement rates between physical and psychological domains following Qigong interventions (44). In this study, Baduanjin and Taichi demonstrated powerful effects on frailty improvement. Previous research has presented that Baduanjin combines stretching and strength training exercises to enhance lower limb strength (45). At the same time, Taichi, focusing on balance and coordination, is notably effective at improving stability in frail older adults (46). Less empirical data concerning Wuqinxi and Yijinjing is available. While most included studies utilised different assessment criteria and intervention methods, and the duration and intensity of interventions varied, these factors likely influenced the degree of frailty improvement. Figure 3’s consistency across studies (all subgroups *p* < 0.001) suggests these factors may be secondary to assessment tool effects. Despite this, various Qigong types demonstrated similar effectiveness in improving frailty. The existing literature suggests that Qigong, including integrated physical exercise, breath control, and psychological regulation, is closely related to their combined effects on physical, psychological, and social health. It supports the argument for their potential significance for multidimensional health management. To improve comparability, future research should accord priority to using internationally recognized (e.g., Fried Phenotype) and pre-register intervention protocols (e.g., standardize movements) to enhance comparability.

### 5.2 Effect on physical ability

#### 5.2.1 Physical performance and daily living activities

The results indicate that TFQs produce significant gains in physical function among frail and pre-frail older adults but do not significantly change activities of daily living. Regarding physical function, three studies (21, 28, 29) utilised the SPPB, which measures balance, gait speed, and chair stand performance to measure mobility, balance, and lower extremity strength (47). The meta-analysis confirms a robust positive effect on this composite measure, with Chen (21) contributing the overwhelming majority (98.1%) of the weight to the pooled estimate. These analyses collectively point to the positive effect of TFQs on these components of physical fitness. The meta-analysis further confirms these results, corroborating previous Baduanjin research that documented increases in lower limb strength through stretching and strength-building exercises (48). Three separate studies (25, 28, 29) utilised different instruments to evaluate improvements in activities of daily living; however, the intervention effects did not reach statistical significance. Notably, while Hou (25) demonstrated a marked improvement (contributing 43.6% weight), the effects reported by Cheung (28) (17.9% weight) and He (29) (38.4% weight) were non-significant, and substantial heterogeneity was present. This contrasts with earlier research and suggest the overall evidence does not confirm the ubiquitous benefit of TFQs for these abilities in frail older adults. The heterogeneity observed is likely attributable to the different measurement scales employed. Perhaps TFQs require greater duration or intensity to generate significant changes in daily activities compared to the more rapid benefits related to physical function (49). The characteristics of TFQs, integrating whole-body movement, breath regulation, and mental focus, likely account for the positive effects on physical function (50). Nevertheless, improvements in activities of daily living necessitate more complicated functional changes that may demand further, more specific interventions aimed at the varied needs of older adults (51). Besides, diverse measurement tools and differing evaluation criteria across the studies add to the range of observed variability. Therefore, future research should precede standardized evaluations to assess the wide-ranging influence of TFQs on frailty.

#### 5.2.2 Balance

The results from this study argue for the positive effects of TFQs on both dynamic and static balance in frail and pre-frail older adults. Subgroup meta-analysis revealed distinct, highly significant effects: dynamic balance (TUG test) showed significant improvement, and static balance (OLST test) also demonstrated significant gains. The extremely high heterogeneity (*I^2^* = 99%) and nonsignificant result (*p* = 0.79) in the initial overall analysis underscore the critical importance of differentiating between these balance types, as confirmed by the highly significant subgroup difference test (*p* < 0.00001, *I^2^* = 99.7%). Enhanced balance is essential for fall prevention, a critical concern for this population. Prior systematic reviews have corroborated the balance-enhancing effects of TFQs in older adults (52). TFQs typically emphasise balance exercises, the key to optimising postural control and visuospatial awareness, which are vital for maintaining stability (53). The deliberate and controlled movements with a traditional nature enable participants to attend to the proprioceptive experience of each posture, thereby optimising sensory perception, motor coordination, and central processing, all of which contribute to better balance (54). This capacity to cultivate a connection between sensory feedback and motor control likely facilitates improved spatial orientation and overall equilibrium. Moreover, the contribution of various Qigong styles on balance differs. This meta-analysis confirms that Tai Chi significantly enhances mobile stability, whereas Wuqinxi and Yijinjing concentrate on mobile and static balance, respectively. In summary, the available data indicate that TFQs, through their integrative approach, play a crucial role in enhancing balance and general physical well-being in older adults.

#### 5.2.3 Grip strength and walking ability

This meta-analysis confirms TFQs significantly enhance upper extremity strength (SMD = 0.76, 95% CI: 0.51–1.02; *I^2^* = 0%) and distance-specific gait metrics in frail older adults, notably enhancing grip strength, gait speed, and ambulation—essential elements for preventing falls and preserving functional autonomy. From a mechanistic standpoint, TFQs improve neuromuscular efficiency via sustained isometric contractions, particularly in upper limb postures such as “Pushing Mountains” from Baduanjin. Such contractions may help counteract age-related reductions in protein synthesis and combat inflammatory muscle degradation (55). The pooled analysis indicated an average 14.1% improvement in grip strength across studies; this aligns with reduced risks of sarcopenia-related disability (56). Regarding lower extremities, TFQs appear to target gait domains differentially. Improvements were observed in short-distance gait speed, representing a clinically meaningful change linked to an 8–10% reduction in fall risk (57). In comparison, the six-minute walking test (6MWT) yielded non-significant gains (pooled MD: 12.47 m, 95% CI: −31.18 to 55.99; *I^2^* = 88%). Task-specific physiological demands could be the reason for this discrepancy. Taichi’s characteristic multidirectional weight-shifting improves rapid force generation (anaerobic capacity), which is beneficial for short bursts of walking. Wuqinxi’s closed-chain exercises, such as “Deer Balancing,” preferentially engage slow-twitch fibers (58); these fibers enhance endurance but might not adequately challenge the aerobic thresholds needed for 6MWT performance. This dissociation between rapid gait gains and endurance limitations reflects distinct neurophysiological pathways: Short-distance improvements primarily engage anaerobic pathways and neuromuscular coordination (59), while sustained walking capacity requires cardiopulmonary adaptation (60). Heterogeneity stemmed from three factors identified through subgroup analyses: (1) the duration of the intervention (comparing 8-week vs. ≥12-week programs), (2) the severity of frailty (pre-frail participants vs. those with moderate frailty), and (3) the specific outcome protocols used (e.g., 4.5 m sprints versus 10 m steady-state walks). Wuqinxi’s focus on strengthening periarticular muscles (61), for instance, improved 10 m gait consistency but had limited transferability to endurance-focused tasks. These observations support the necessity for standardized TFQs protocols that are stratified according to frailty level and specific targeted outcomes (e.g., power versus endurance). TFQs remain a safe exercise option for older adults for whom high-intensity training is contraindicated, despite these identified limitations. Future interventions designed to address endurance deficits could potentially integrate TFQs with task-specific training. An example would be combining the flexibility sequences of Baduanjin with interval walking, aiming to synergistically enhance aerobic capacity and improve fall prevention (62).

### 5.3 Effect on cognitive function

This meta-analysis shows that in older persons with frailty or pre-frailty, TFQs considerably improve cognitive performance. The strong and consistent character of these findings suggests that TFQs techniques can improve cognitive ability, maybe by means of their integrated mind–body approach. TFQs emphasise mental concentration and regulation of breathing, factors known to contribute to enhanced cognitive reserve and neuroplasticity (63). The domain-specific improvements observed (Section 3.6) particularly highlight enhanced cognitive mobilisation – the capacity to efficiently allocate attentional resources during goal-directed tasks. This manifests as improved executive planning, evidenced by Tsang’s LOTCA-G results (*p* < 0.01) (26). Critically for pre-frail older adults, this mobilisation operates as an arousal catalyst (64), priming engagement and alertness essential for movement planning. Physical activity that is part of TFQs may benefit brain health by reducing inflammatory markers, promoting cerebral blood flow, and facilitating synaptic plasticity, and is further reinforced by restorative attention effects where mental effort expenditure is reduced during sustained concentration (65), forming a cyclical rest-activity dynamic (66) where attentional engagement energises cognitive-physical integration. Moreover, the mental engagement necessary for acquiring and executing the choreographed movements of Qigong exercises likely stimulates executive cognitive processes crucial for planning, memory, and problem-solving. Further corroborating these findings, Tsang (26) documented significant improvements using the Loewenstein Occupational Therapy Cognitive Assessment-Geriatric LOTCA-G which specifically captures executive planning abilities to evaluate cognitive function. These mechanisms align with the cognitive-energetic framework (64) wherein mind–body integration optimizes prefrontal resource allocation, particularly vital for pre-frail individuals whose functional decline originates from disrupted cognition-motor coupling This work supports the conclusion that TFQ practices can enhance cognitive abilities, as measured by various cognitive assessment instruments.

### 5.4 Effect on quality of life

These findings demonstrate that TFQs significantly benefit frail and pre-frail older adults, improving their quality of life, mood, and sleep. These improvements indicate that TFQ is well-suited to address this vulnerable population’s physical and psychological needs.

The advantages of TFQs derive from their gentle integration of movement, breathing exercises, and mental focus. In contrast to exercise regimens focused solely on physical fitness, TFQs enhance physical characteristics such as balance and mobility while simultaneously cultivating mental relaxation and emotional relief through its holistic mind–body approach (67). Group practice of TFQs cultivates a sense of community, reduces loneliness, and can cultivate emotional support networks—all vital for promoting mental well-being in older adults. TFQs practice may also reduce negative emotions by influencing the autonomic nervous system, specifically by increasing parasympathetic activity. Such mind–body practices involve regulated breathing and improved vagal tone, which is associated with decreased anxiety and depression (68). The convergence of QoL enhancement (SMD = 1.17) and negative emotion reduction (SMD = −0.79) can be interpreted through a unified cognitive-energetic lens (69): By mitigating emotional drain (e.g., GDS/HAMD scores) and boosting cognitive resources, TFQs likely free mental capacity for engagement in meaningful activities, thereby amplifying perceived life quality. This is corroborated by the strong association between emotion regulation and SF-36 vitality scores [r = 0.62 in Chen (21)].

Sleep disruptions frequently affect older adults. In contrast to pharmaceutical approaches, TFQs offer low-impact movements, rhythmic breathing, and stress reduction, potentially improving sleep quality by enhancing circadian rhythms. Combining physical activity and mental relaxation contributes to overall well-being, positively affecting sleep. Improved sleep quality (SMD = 1.28) may further sustain cognitive-energetic gains, as restorative sleep is posited to reset attentional capacity and consolidate emotional regulation – synergistically supporting daytime functioning (70). Despite these encouraging results, a deeper understanding of the differential effects of different TFQs forms is needed. For instance, specific modalities such as Baduanjin, Taichi, and Wuqinxi could each offer unique advantages—whether in promoting balance, muscle strength, or emotional well-being. More research is necessary to determine which Qigong types benefit specific health outcomes. Future research should also consider standardising assessment instruments to fully reflect the wide-ranging benefits of TFQ interventions, enabling researchers to offer more individualised and targeted guidance.

### 5.5 Traditional fitness qigong exercise program

This review synthesises evidence from multiple studies on Qigong interventions, focusing on how the duration, frequency, setting, and type of Qigong practice affect frailty outcomes in older adults. Intervention duration varied widely, from as brief as 2 weeks (29) to as long as 48 weeks (34), with longer programs (≥12 weeks) generally producing more reliable improvements in physical performance, balance, and psychological well-being (23, 30). The typical frequency was three to five sessions per week; while higher frequencies (e.g., daily practice) might produce quicker results, they can also create adherence challenges for frail older adults (22, 28). Most interventions occurred in community or institutional settings to facilitate professional oversight (26, 38). However, home-based practices such as simplified Tai Chi and newspaper-guided Wuqinxi improved access for participants with limited mobility (22, 24). Regarding the Qigong type, Baduanjin was the most frequently evaluated and consistently compelling, indicating significant advantages for physical function and psychological health across several trials (25, 31, 32). Tai Chi also produced robust gains in balance, quality of life, and frailty indices, especially in programs of longer duration (34, 36). While less frequently assessed, both Wuqinxi and Yijinjing positively affect mental health and physical capacity (38, 39). Crucially, these benefits may arise partly through enhanced corticomuscular coupling, which compensates for neural deficits by improving postural correction and leveraging attention restoration during mindful movement (71). These findings demonstrate that TFQs provide dual value for frail older adults: 1. Low-barrier accessibility through culturally meaningful formats adaptable to diverse settings (72); 2. Energetic mobilisation linking cognition to movement execution, fostering functional resilience as conceptualized in behavioral engagement theories (73). These findings suggest that implementing TFQs tailored to specific needs, optimising them for adherence and effectiveness, with prioritization of consistent long-term practice (≥12 weeks, 3–5 sessions/week) and culturally accessible forms, represents an evidence-based strategy to actively counteract frailty through neural and cognitive-motor adaptation (74).

## 6 Limitations and potential directions for future research

Though it makes some contributions, this meta-analysis has certain limits. Initially, the predominance of English and Chinese publications could cause selection bias. Second, if studies with low means and standard deviations had been eliminated, the outcomes might have been different. Crucially, substantial variability in intervention methodologies and, importantly, in the specific assessment instruments used across the included studies resulted in pronounced heterogeneity in specific outcomes; This variability reflects a broader methodological constraint: as noted by Guyatt et al. (75), while all included studies were randomised trials, most represent Type 2 evidence (group comparisons with passive controls such as usual care) due to the absence of active comparator groups or standardized manipulation protocols. Consequently, instrument variability (where different tools may measure constructs differently, have varying sensitivity, or use incompatible metrics) complicates direct comparisons and potentially affects the precision, interpretability, and broader generalisability of the pooled effect estimates. Moreover, differences in intervention length and specific strategies employed may affect the observed effects’ stability; for instance, longer interventions may produce more significant results. While sensitivity analyses (including model transformation and sequential study omission) supported the robustness of the main findings, a formal assessment of publication bias (e.g., via funnel plots or Egger’s regression test) was not feasible for the primary pooled estimates or most subgroup analyses due to the limited number of studies available per specific outcome or subgroup (typically fewer than 10); consequently, the possibility remains that the overall findings could be influenced by an over-representation of studies with statistically significant positive results, while smaller studies or those with null/negative findings might be underrepresented, particularly within specific outcome domains. Therefore, future work should prioritise appraising the psychometric properties and comparability of statistical instruments used in TFQ research, explicitly comparing interventions of varying lengths to understand better their long-term effects in older adults with frail or pre-frail conditions and, where sample sizes permit, formally assessing publication bias in future syntheses, while also advancing toward Type 3–4 evidence through actively controlled trials with standardized fidelity protocols (e.g., sham procedures/attention-matched controls) to establish causal specificity.

## Data Availability

The original contributions presented in the study are included in the article/supplementary material, further inquiries can be directed to the corresponding author.
